# Comparison of coronary sinus diameter Z-scores in normal fetuses and fetuses with persistent left superior vena cava (PLSVC)

**DOI:** 10.1007/s10554-017-1229-5

**Published:** 2017-08-14

**Authors:** Mingming Ma, Yan Tan, Ran Chen, Yankai Mao, Bei Wang, Bowen Zhao

**Affiliations:** Department of Diagnostic Ultrasound and Echocardiography, Sir Run Shaw Hospital, Zhejiang University College of Medicine, No.3 East Qingchun Road, Hangzhou, 310016 China

**Keywords:** Fetal echocardiography, Coronary sinus, Persistent left superior vena cava, Noncardiac fetal biometric parameters, Z-scores

## Abstract

To establish Z-score reference ranges for coronary sinus (CS) diameter in normal fetuses and explore the diagnostic value of CS Z-score in fetuses with persistent left superior vena cava (PLSVC). Study of 235 normal fetuses and 30 fetuses with PLSVC was involved. Noncardiac biometrical parameters included biparietal diameter (BPD), femoral length (FL), heart area (HA), gestation age (GA). The coronary sinus systolic and diastolic diameter (CSDs and CSDd ) were measured at the end of systole and diastole. CSDs and CSDd Z-score models were constructed by using linear regression analysis with Non-cardiac biometrical parameters as independent variables. Z-scores between normal fetuses and fetuses with PLSVC were compared. A simple, linear regression model was the best description and correlations between fetal CSDs and CSDd and four independent variables were excellent. Reference ranges for predicting means and SDs of the fetal CS were established. Equations for Z-score calculation were provided, CSDs and CSDd Z-scores were statistically different between normal fetuses and those with PLSVC. Development of CSDs and CSDd Z-score reference ranges in normal fetuses was realized. The CSDs and CSDd Z-scores can provide quantitative evidence in prenatal diagnosis of PLSVC.

## Introduction

The coronary sinus (CS) lies adjacent and slightly posterior to the mitral valve annulus within the left atrioventricular groove. The CS enters the right atrium below the level of the foramen ovale just above the valve of the inferior vena cava and is the main coronary venous return [1, 2]. In some instances, the CS is dilated due to volume overload or more rarely to pressure overload. The persistent left superior vena cava (PLSVC) typically drains into the right atrium via the dilated CS and represents the most common cause of CS dilation. Given that amniotic fluid and fetal lungs serve as a good acoustic window during the second and third trimesters of pregnancy and given the improved resolution power of modern ultrasound equipment, relatively satisfactory echocardiographic images of the CS can be obtained in most of the fetus. The CS can be visualized from the apical or basal four-chamber view, but the apical four-chamber view is the most commonly used.

Recently, reliable Z-scores have been developed for fetal echocardiographic parameters based on fetal biometric measurements or gestational age (GA), allowing better quantification of how much an individual’s measurement lies above or below the expected value [3].

To the best of our knowledge, reference ranges for qualitative assessment of CS diameter derived from M-mode fetal echocardiography of normal fetuses and PLSVC fetuses based on a large sample size have not been previously reported. We undertook this study to establish normal reference ranges of the Z-score of CS diameter in normal fetuses during the second and third trimesters of pregnancy using the methodology introduced by Altman et al. [4] and Royston et al. [5] and to explore the diagnostic value of CS Z-score in fetuses with PLSVC.

## Materials and methods

### Study population

Two groups of fetuses were identified. The first group consisted of 235 singleton pregnancies undergoing routine pregnancy ultrasound scans from December 2014 to January 2015. The fetuses were scanned from the 20th to 40th week of gestation. The inclusion criteria were as follows: (1) singleton pregnancies, (2) absence of fetal cardiac and extracardiac abnormalities, (3) GA based upon regular menstruation corroborated by early ultrasonic measurement of the crown-rump length, and (4) the presence of a standard view. The criteria for exclusion were as follows: (1) maternal medical complications, such as diabetes mellitus, hypertension, immune or renal disease; and (2) chromosomal abnormalities. These subjects were also used as the control group for comparison with the study group, comprising fetuses with a prenatal diagnosis of PLSV. The ethical committee approved the study protocol and all pregnant women provided informed consent to participate.

The study group consisted of 30 fetuses with PLSVC at echocardiography recruited from December 2014 to January 2015 whose examinations were conducted between the 22nd and 39th weeks of gestation. The diagnosis of fetal PLSVC was confirmed by postnatal echocardiography.

### Echocardiographic examinations

**Fig. 1:**
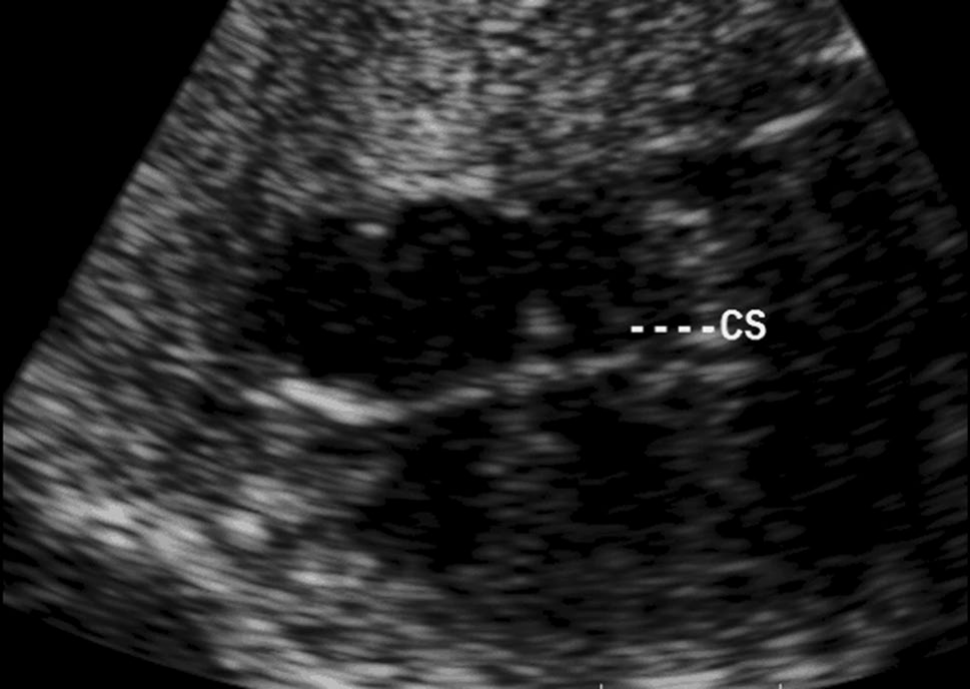
The fetal heart is imaged in a basic four-chamber view. The wall of the coronary sinus can be visualized

**Fig. 2:**
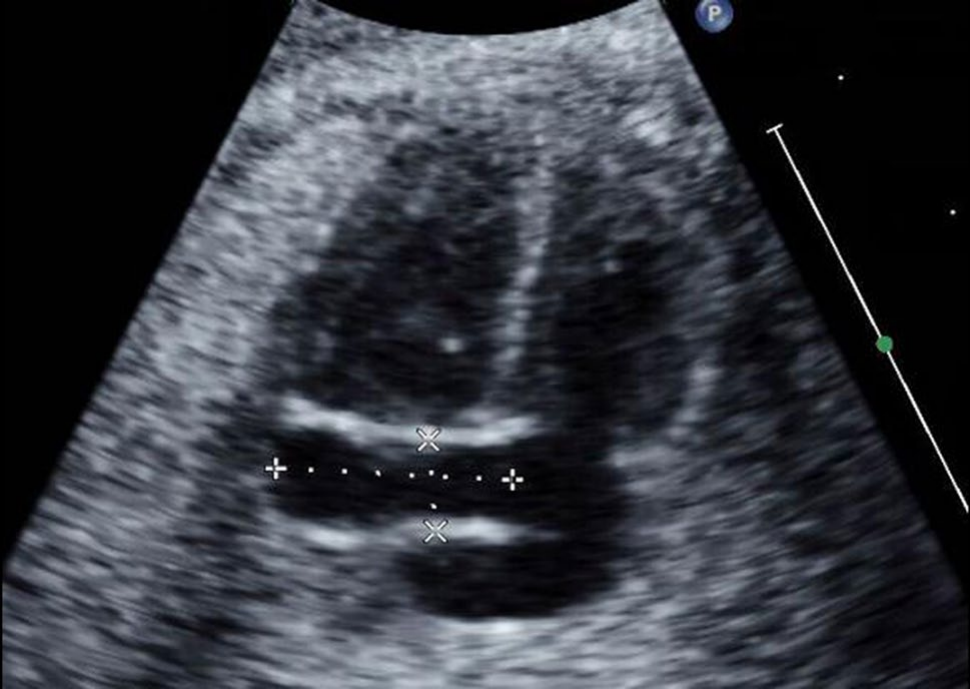
A standard fetal apical four-chamber view. Dilatation of the coronary sinus with PLSVC can be visualized

**Fig. 3:**
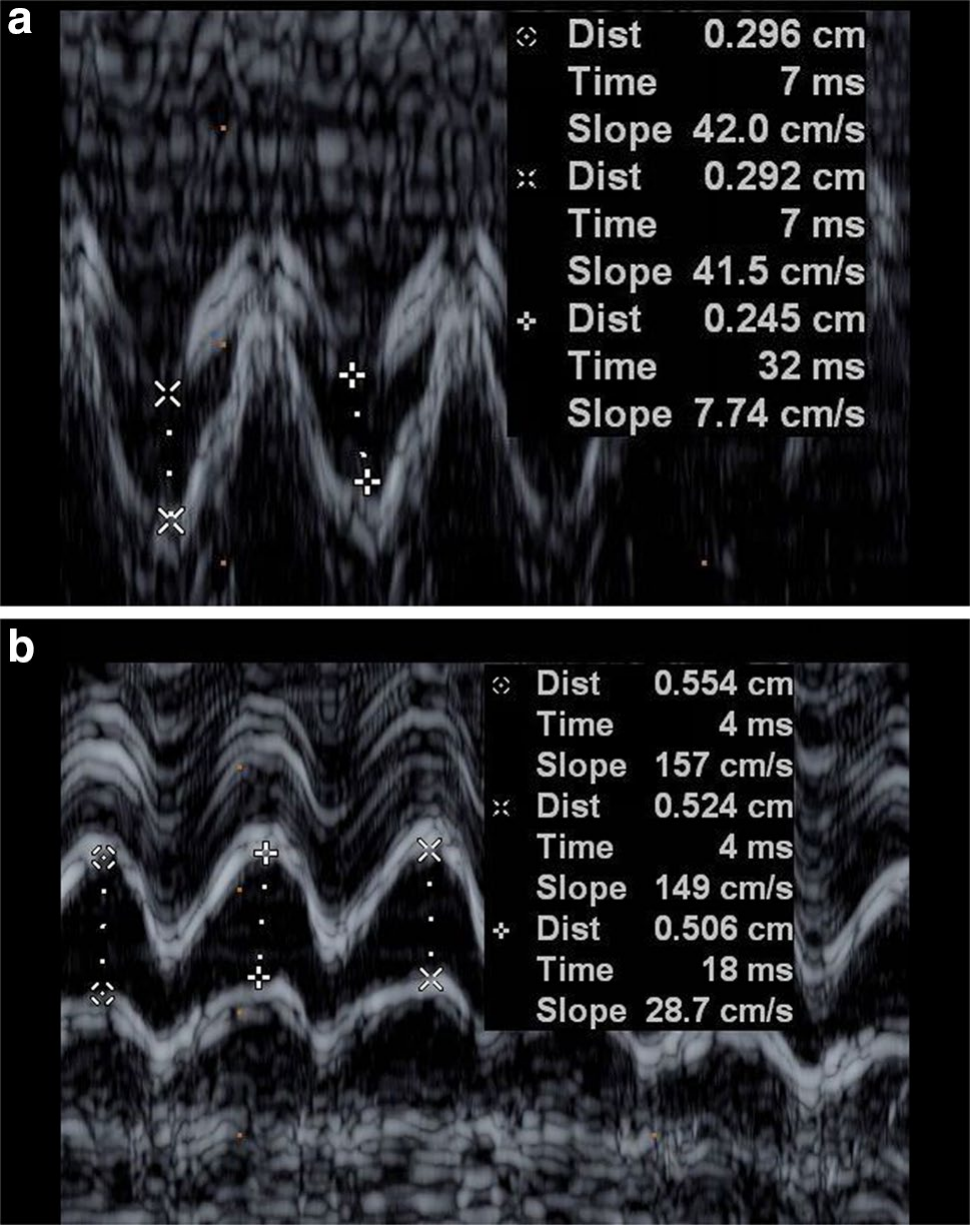
**a** M-mode tracing obtained from a normal coronary sinus. **b** M-mode tracing obtained from a dilated coronary sinus with PLSVC

All fetuses underwent echocardiographic examinations by two well-trained echocardiographers using a Philips iE33 ultrasound system (Philips, Andover, Mass, USA) with transducers of 5 − 1 and 8 − 3 MHz. Fetal echocardiographic studies were performed from standard scanning planes of the apical four-chamber view or the basal four-chamber view. After magnification of the CS near the drainage site into the right atrium (Figs. 1, 2), the M-Mode cursor was placed on the middle portion of both walls of the CS. Maximum systolic and diastolic diameters were measured using M-mode echocardiography (Fig. 3a, b). All patients underwent fetal biometric measurements with an anatomic survey including femur length (FL), biparietal diameter (BPD), heart area (HA) and GA. GA was calculated from the date of the last menstrual period. If appropriate, sonographic age was used to adjust menstrual age when a discrepancy was noted between size and dates. Three separate measurements were obtained in each case, and the mean value was recorded for analysis. If more than one measurement of a fetus was performed during a pregnancy, then only one of the values for each fetus was randomly selected for the reference sample.

### Statistical analysis

SPSS package 19.0 (SPSS, Inc., Chicago, IL, USA) was used to perform the statistical analyses following the methodology outlined by Royston and Wright [5] to determine the optimal model and Z-scores for reference ranges of CS. Briefly, using CSDs and CSDd as the dependent variables and GA, FDL, HA and BPD as independent variables, regression analysis of the mean and SDs were performed, comparing linear, quadratic, cubic and logarithmic models to identify the optimal model. The best-fitted models for the SD were derived from regression of scaled absolute residuals, which were obtained as: 1.25 × absolute (measured value − estimated value). Then, the Z-score was expressed as (observed value − predicted value)/predicted SD. The Shapiro–Wilk W test for normality of the Z*-*scores was applied to determine whether the values conformed to a normal distribution. If needed, statistical transformation was performed. Z-scores for PLSVC were calculated based on the constructed statistical models.

The mean and SD of the Z-scores were calculated for each variable in each group, and the differences were compared using Student’s t-test. *P*-values of <0.05 were considered statistically significant.

## Results

A total of 265 subjects were recruited for this study. In eight of these cases, the CS could not be accurately visualized. Thus, a total of 257 subjects were ultimately included. Of these, 227 cases had normal CS (group 1), whereas 30 had dilated CS (group 2). The mean GA was 29 + 4 weeks for group 1 and 28 + 3 weeks for group 2. The mean age of pregnant women was 28.68 ± 4.07 years for group 1 and 28.00 ± 5.54 years for group 2.

The apical four-chamber view was utilized for measurement in 185 (71.9%) fetuses for final analysis. In 72 (28.1%) fetuses, the basal four-chamber view was used owing due to an anterior position of the fetal spine. Fetal CS measurements were successfully obtained in all of these cases.

**Fig. 4:**
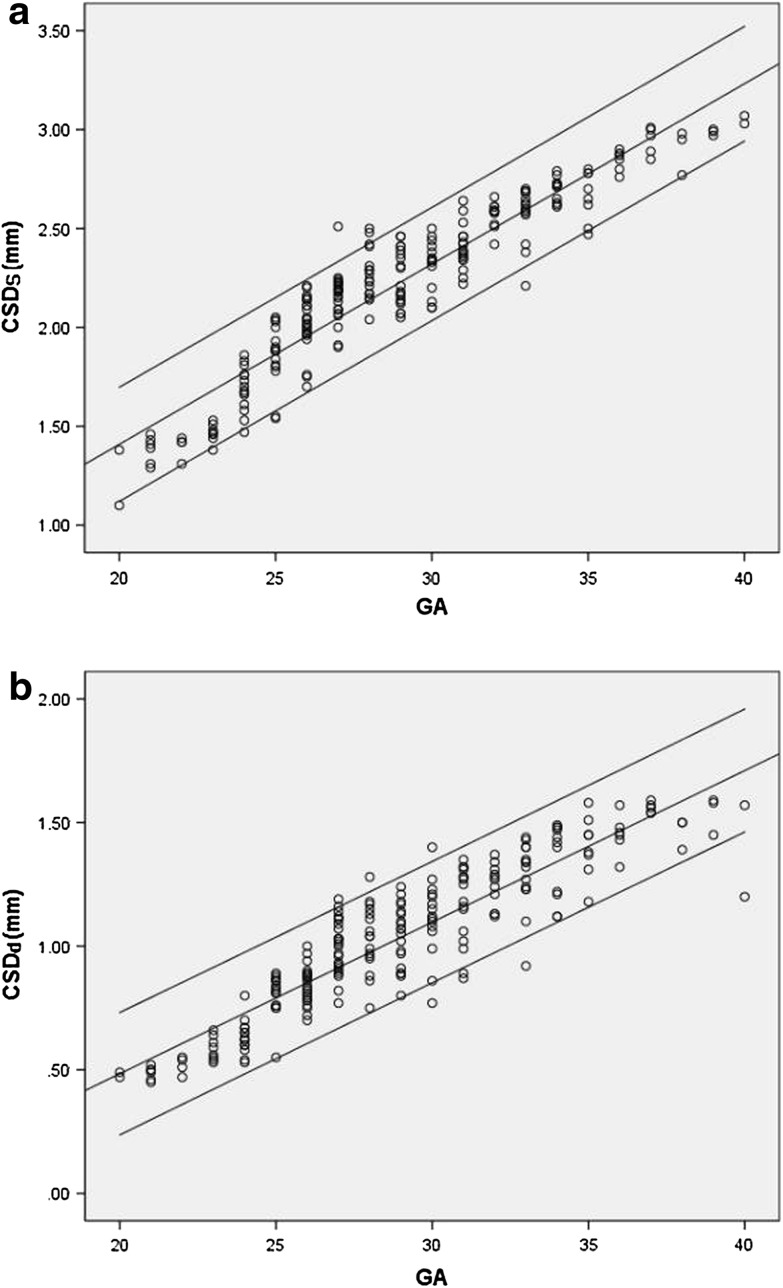
**a** A scatter plot of the CSDs based on GA, and the 5th, 50th and 95th percentiles are superimposed. **b** A scatter plot of the CSDd based on GA, and the 5th, 50th and 95th percentiles are superimposed

**Fig. 5:**
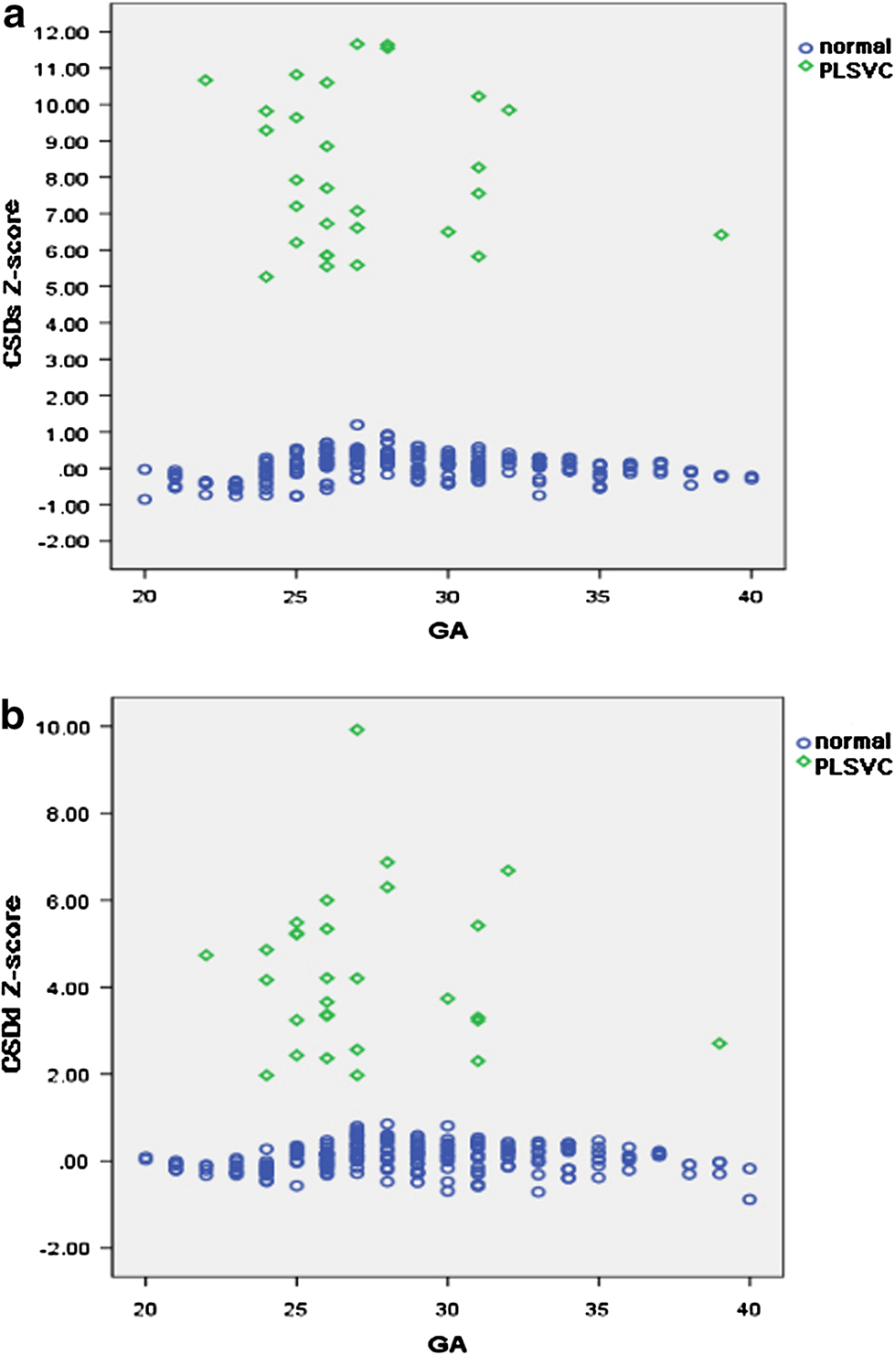
**a** CSDs Z-scores against GA in fetuses with PLSVC and normal fetuses. **b** CSDd Z-scores against GA in fetuses with PLSVC and normal fetuses

The diameter of the CS derived from conventional M-mode echocardiography increased with the duration of the gestation and advancing HA. Correlations between fetal CSDs and CSDd and four independent variables (BPD, FL, HA and GA) were excellent. GA had strongly correlation with CSDs and CSDd (*r* = 0.940 and 0.908, all *P* < 0.001). The best-fitted regression equations, correlation coefficients, *P*-values of the mean, and SD of the CSDs and CSDd against BPD, FL, HA and GA are presented in Tables 1 and 2. The linear regression equations were the best-fitted models for the mean and the SD. The adequacy of these statistical models was validated by developing a Z-score for each variable. Figure 4a, b present a scatter plot of CSDs and CSDd values based on GA, and the 5th, 50th and 95th percentiles are superimposed. The normative fetal CSDs and CSDd percentile charts are also presented in Table 3. The Z-score for the CSDs and CSDd against GA was computed based on the linear mean and SD. The distribution of the Z-scores suggested a Gaussian distribution. Additionally, Z-scores were evenly distributed above and below 0 across the entire range of GAs and exhibited a standard normal distribution. Z-scores that were outside the limits did not significantly differ from the expected 10% of the scores. A normal distribution was demonstrated with the Shapiro–Wilk W test. All of the regression statistical models fit the data well. Table 4 summarizes Z-scores of the CSDs and CSDd in each group. Z-scores in the PLSVC group differed from those of the normal group by >2 Z-scores compared with mean Z-scores between the two groups as assessed by Student’s t-test. Compared with the normal group, the mean CSDs and CSDd *Z*-scores were significantly increased in the PLSVC group (*P* < 0.001). The cardiovascular parameters in the PLSVC and normal groups were plotted against GA (Fig. 5a, b).

**Table 1:** Regression analysis of CSDs and CSDd based on FL, BPD, HA and GA

Parameters	Regression equation	r(P)
FL, cm		
CSDs, cm	0.003 + (0.041 × FL)	0.895 (<0.001)
CSDd, cm	−0.044 + (0.028 × FL)	0.865 (<0.001)
BPD, cm		
CSDs, cm	−0.031 + (0.034 × BPD)	0.897 (<0.001)
CSDd, cm	−0.067 + (0.023 × BPD)	0.870 (<0.001)
HA, cm^2^		
CSDs, cm	0.133 + (0.010 × HA)	0.849 (<0.001)
CSDd ,cm	0.042 + (0.007 × HA)	0.838 (<0.001)
GA, weeks		
CSDs, cm	−0.041 + (0.009 × GA)	0.940 (<0.001)
CSDd, cm	−0.074 + (0.06 × GA)	0.908 (<0.001)

*FL* femur length, *BPD* biparietal diameter, *HA* heart area, *GA* gestational age, *CSDs* coronary sinus systolic diameter, *CSDd* coronary sinus diastolic diameter

**Table 2:** Regression analysis of the SD of CSDs and CSDd based on FL, BPD ,HA and GA

Parameters	Regression equation	r(P)
FL, cm		
SD of CSDs, cm	0.014 + (0.005 × FL)	0.579 (<0.001)
SD of CSDd, cm	0.011 + (0.004 × FL)	0.590 (<0.001)
BPD, cm		
SD of CSDs, cm	0.018 + (0.005 × BPD)	0.580 (<0.001)
SD of CSDd, cm	0.014 + (0.004 × BPD)	0.6111 (<0.001)
HA, cm^2^		
SD of CSDs, cm	0.043 + (0.006 × HA)	0.628 (<0.001)
SD of CSDd, cm	0.031 + (0.004 × HA)	0.640 (<0.001)
GA, weeks		
SD of CSDs, cm	0.014 + (0.001 × GA)	0.534 (<0.001)
SD of CSDd, cm	0.012 + (0.001 × GA)	0.561 (<0.001)

*FL* femur length, *BPD* biparietal diameter, *HA* heart area, *GA* gestational age, *CSDs* coronary sinus systolic diameter, *CSDd* coronary sinus diastolic diameter, *SD* standard deviation

**Table 3:** The mean, 5th and 95th percentiles of CSDs and CSDd against FL, BPD ,HA and GA

Parameter (cm)	FL	BPD	HA	GA
CSDs	0.221 (0.135–0.307)	0.215 (0.128–0.303)	0.240 (0.157–0.323)	0.229 (0.139–0.319)
CSDd	0.105 (0.046–0.164)	0.105(0.046–0.164)	0.117(0.059–0.170)	0.106 (0.046–0.166)

*FL* femur length,*BPD* biparietal diameter, *HA* heart area, *GA* gestational age, *CSDs* coronary sinus systolic diameter, *CSDd* coronary sinus diastolic diameter

**Table 4:** Comparison of parameters between fetuses with PLSCV and normal fetuses

Parameter	PLSVC(n = 30)	Normal (n = 227)	P
FL			
Z-score of CSDs	9.265 ± 3.792	0.034 ± 1.512	<0.0001
Z-score of CSDd	8.185 ± 4.951	0.007 ± 1.304	<0.0001
BPD			
Z-score of CSDs	7.734 ± 3.265	0.014 ± 1.182	<0.0001
Z-score of CSDd	5.712 ± 3.402	0.018 ± 0.881	<0.0001
HA			
Z-score of CSDs	3.816 ± 1.889	−0.111 ± 0.721	<0.0001
Z-score of CSDd	3.541 ± 2.182	0.035 ± 0.654	<0.0001
GA			
Z-score of CSDs	3.198 ± 8.461	0.175 ± 1.081	<0.0001
Z-score of CSDd	6.385 ± 3.539	−0.013 ± 0.908	<0.0001

Data are given as mean ± SD

*FL* femur length, *BPD* biparietal diameter, *HA* heart area, *GA* gestational age, *CSDs* coronary sinus systolic diameter, *CSDd* coronary sinus diastolic diameter

## Discussion

PLSVC is an embryological remnant that represents persistence of the embryonic left anterior cardinal vein and is the most common form of anomalous systemic venous return. The condition is noted in 0.3% of postmortems in healthy individuals and up to 4–8% in patients with congenital heart disease [6]. The initial description of this anomaly was provided by Edwards and Du Shane in 1950 [7]. The condition has no clinical signs and is typically discovered incidentally. However, the condition is associated with a high incidence of accompanying congenital heart defects, such as atrial septal defects, anomalous pulmonary venous connections, endocardial cushion defect and Tetralogy of Fallot [8]. Furthermore, PLSVC can typically pose difficulties with venous catheterization, pacemaker implantation and coronary artery bypass graft surgery [9, 10]. In addition, the condition is also associated with an increased incidence of arrhythmias and conduction disturbances. Thus, a prenatal diagnosis of PLSVC plays an important role in prenatal counseling and management.

Traditional centiles ranges have been compiled by previous investigations based on menstrual age (MA) to estimate the size and growth of fetal cardiac structures [11, 12]; however, this approach poses challenges for values that vary from the norm. A more effective alternative approach to centiles is the use of Z-scores. Fetal Z-score models have been proposed by numerous investigators and have been increasingly used in recent years [5, 13]. According to these models, the Z-score is an expression of the number of SD measurements above or below the mean value for a given population, allowing the accurate quantification of the growth of cardiac structures. The *Z*-score represents a numerical value that clinicians can easily interpret. In any normal distribution, 68% of the population would be classified within its mean ± 1SD (Z = ±1), 95% within mean ± 2SD (Z = ±2), and 99% within ± 3SD (Z = ±3) [14]. The use of Z-scores permits a more precise assessment of cardiac dimensions.

To date, evaluation of the CS is primarily achieved by gray-scale, M-mode echocardiography and Doppler sonography. Reference values of the CS diameter have been reported for fetuses in a few studies, Most of these studies used GA as the only fetal size parameter. Our study and previous results have consistently developed a linear relationship between GA and the CS diameter. Rein et al. [1] demonstrated that the normal CS diameter correlated well with the GA and increases over the course of gestation. Abello et al. [2] demonstrated that the CS diameter increases in a linear fashion with GA in both systole and diastole, corresponding to fetal cardiac growth. Our results demonstrated that the CS diameter increased with GA, BPD, HA and FL. Our reference values for fetal CSDs and CSDd differed slightly compared with those previously reported. The difference is presumably due to the variation in echocardiographic techniques. Rein et al. established normal values for CS dimensions using gray-scale echocardiography during gestation. The diameter ranged from 1 to 3.2 mm in normal fetuses. Previous investigators have suggested that measurement of the CS diameter should be related to cardiac cycle [15]; however, Rein et al. did not correlate the measurement to the phase of the cardiac cycle because measurements were obtained from the frozen image when gray-scale echocardiography was used. This feature may pose a challenge to determine the phase of the cardiac cycle in which the image was obtained. Similarly, when measurements were acquired from the cineloop, certain images may overlap, depending on the frame rate at which the examination was implemented. Either of these factors would probably have an effect on the precision of measurements. Abello et al. and our study used M-mode echocardiography to measure CS diameters in both systole and diastole to overcome the disadvantage of using gray-scale echocardiography. Both CSDs and CSDd values obtained by our study were slightly reduced compared with those obtained by Abello et al. (CSDs: 1.4–3.2 vs. 1.6–4.0 mm; CSDd: 0.5–1.6 vs. 0.9–2.2 mm). Variations in nationality and sample size may have contributed to the discrepancies. In addition, our investigation demonstrated that CS diameters of PLSVC fetuses were approximately three times larger compared with normal fetuses (CSDs: 3.7–7.0 vs. 1.4–3.2 mm; CSDd: 1.4–4.8 vs. 0.5–1.6 mm), which is consistent with previously reported data [1].

To our best knowledge, reference ranges for normal fetal CSDs and CSDd Z-scores have not been reported previously. Schneider et al. [13] first introduced cardiac dimension Z-score models for non-cardiac fetal biometry instead of GA. They suggested that in cases where the GA cannot be precisely calculated, other accessible parameters, such as BPD or FL, could be used to overcome the disadvantage of using the GA as the only fetal size parameter [16]. In this study, the normal range for CSDs and CSDd based on HA are also provided, and the statistical model was constructed based on HA. Our results demonstrate that CS diameters increased with the GA, BPD, HA and FL; moreover, good correlations were noted for the various parameters. Rein et al. [1] demonstrated that the CS diameter of these abnormal fetuses with PLSVC was at least four Z-scores greater than the estimated normal value at any age of gestation, which is similar to our findings. Our investigation demonstrated that the CSDs and CSDd Z-scores of PLSVC fetuses are significantly increased compared with normal fetuses. Most of the CSDs and CSDd Z-scores are greater than +2. CSDd and CSDs Z-scores of less than 2 were observed in some fetuses in the PLSVC group using HA as an independent variable, but comparison of CSDd and CSDs Z-scores between the two groups demonstrated significant differences using t-tests.

This study had several limitations. First, this study provides information limited to 20–40 weeks of gestation, and we were unable to monitor all of the fetuses to term. Second, the inter-observer and intra-observer variability of the two operators’ measurements were not assessed. Another limitation is that none of the newborns in the normal group underwent detailed echocardiography.

## Conclusions

In summary, the calculation of CSDd and CSDs Z-scores that we developed allows simple, effective, and accurate evaluation of CS dimensions and provides a quantitative basis for the prenatal diagnosis of PLSVC. However, the effectiveness of these fetal parameters should be validated by further investigations.
